# Unraveling the architecture of the dorsal raphe synaptic neuropil using high-resolution neuroanatomy

**DOI:** 10.3389/fncir.2014.00105

**Published:** 2014-08-26

**Authors:** Mariano Soiza-Reilly, Kathryn G. Commons

**Affiliations:** ^1^Institut du Fer à Moulin, INSERM, UMR-S 839Paris, France; ^2^Université Pierre et Marie CurieParis, France; ^3^Department of Anesthesiology, Perioperative, and Pain Medicine, Boston Children’s HospitalBoston, MA, USA; ^4^Department of Anaesthesia, Harvard Medical SchoolBoston, MA, USA

**Keywords:** vesicular glutamate transporter, glutamate decarboxylase 65, synapses, ultrathin serial-sections, axo-axonic

## Abstract

The dorsal raphe nucleus (DRN), representing the main source of brain’s serotonin, is implicated in the pathophysiology and therapeutics of several mental disorders that can be debilitating and life-long including depression, anxiety and autism. The activity of DRN neurons is precisely regulated, both phasically and tonically, by excitatory glutamate and inhibitory GABAergic axons arising from extra-raphe areas as well as from local sources within the nucleus. Changes in serotonin neurotransmission associated with pathophysiology may be encoded by alterations within this network of regulatory afferents. However, the complex organization of the DRN circuitry remains still poorly understood. Using a recently developed high-resolution immunofluorescence technique called array tomography (AT) we quantitatively analyzed the relative contribution of different populations of glutamate axons originating from different brain regions to the excitatory drive of the DRN. Additionally, we examined the presence of GABA axons within the DRN and their possible association with glutamate axons. In this review, we summarize our findings on the architecture of the rodent DRN synaptic neuropil using high-resolution neuroanatomy, and discuss possible functional implications for the nucleus. Understanding of the synaptic architecture of neural circuits at high resolution will pave the way to understand how neural structure and function may be perturbed in pathological states.

## The complex architecture of the DRN

The dorsal raphe nucleus (DRN) comprises the majority of cells in the brain with the capacity of synthetizing the neurotransmitter serotonin (5-hydroxytryptamine, 5-HT) (Steinbusch, 1984). These cells represent the main source of 5-HT for forebrain regions, in which 5-HT regulates the activity of local networks. In agreement with its widespread projection targets, the DRN 5-HT system is involved in a broad repertoire of physiological processes. These include the modulation of motivational states, stress, appetite, sleep, and aggression, among others (Jacobs and Azmitia, 1992; Amat et al., 2005; Nakamura et al., 2008; Monti, 2010; Takahashi et al., 2010; Bruchas et al., 2011; Rozeske et al., 2011; Warden et al., 2012). Additionally, the DRN 5-HT system has been implicated in the pathophysiology and therapeutics of mental disorders such as depression, anxiety, autism, etc…(Stockmeier, 1997; Arango et al., 2001; Bach-Mizrachi et al., 2006, 2008; Bruchas et al., 2011; Matthews and Harrison, 2012; Kerman et al., 2012; Veenstra-VanderWeele et al., 2012). Dysfunction of 5-HT neurotransmission associated with these disorders may reflect alterations in axonal networks and synaptic circuits within the DRN.

**Figure 1:**
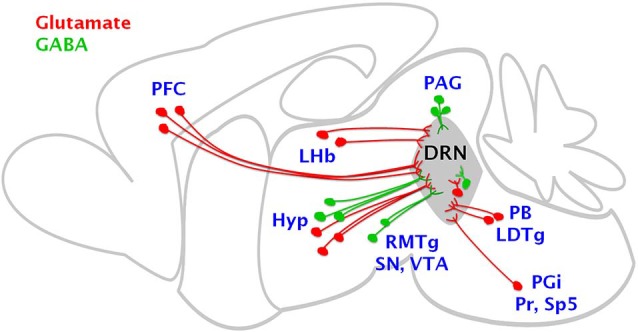
**Brain regions sending glutamate and GABA axon projections to the DRN**. The cartoon summarizes the main sources of glutamate excitatory and GABAergic inhibitory inputs to the DRN. Glutamate cells (in red) are mainly located in the prefrontal cortex (PFC), lateral habenula (LHb), hypothalamus (Hyp), parabrachial nucleus (PB), laterodorsal tegmental nucleus (LDTg), paragigantocellular nucleus (PGi), prepositus hypoglossal nucleus (Pr) as well as the spinal trigeminal nucleus (Sp5), as well as in local sources including the DRN. GABAergic cells (in green) sending projections to the DRN are located mainly in the hypothalamus, substantia nigra (SN), ventral tegmental area (VTA), rostromedial tegmental nucleus (RMTg) and locally within the periaqueductal gray (PAG) and DRN.

The activity of DRN neurons is finely modulated by the complex interaction of glutamatergic excitatory and GABAergic inhibitory neurotransmission mediated by glutamate and GABA axons arising from extra-raphe areas as well as from local sources (Figure 1). Glutamatergic axons originating from extra-raphe areas include the hypothalamus, lateral habenula and prefrontal cortex (Kalén et al., 1985; Lee et al., 2003; Sego et al., 2014) as well as from local sources (Jolas and Aghajanian, 1997; Commons, 2009; Hioki et al., 2010). More caudally, several medullary regions send glutamate projections to the DRN including the parabrachial nucleus and the laterodorsal tegmental nucleus, and to a lesser extent, the paragigantocellular nucleus, the prepositus hypoglossal nucleus as well as the spinal trigeminal nucleus (Lee et al., 2003; Figure 1). Furthermore, different populations of glutamatergic neurons sending axon projections to the DRN have a preferred expression of specific types of the vesicular glutamate transporter (VGLUT1, VGLUT2 and VGLUT3), since there is a topographic segregation of cells expressing these different types of transporters (reviewed by Soiza-Reilly and Commons, 2011a). These proteins have the capacity of filling the synaptic vesicles with glutamate and their identification represents a very useful tool to identify different types of glutamate axons. This analysis allowed us to examine the relative contribution of different populations of neurons, originating in different brain regions, to glutamate excitatory drive of the DRN (Soiza-Reilly and Commons, 2011b).

GABAergic axons innervating the DRN originate from extrinsic sources including the lateral and rostral hypothalamic and preoptic areas, substantia nigra, ventral tegmental area (Gervasoni et al., 2000; Kirouac et al., 2004; Taylor et al., 2014), and the rostromedial tegmental nucleus (Lavezzi et al., 2012; Sego et al., 2014; Figure 1). Local GABAergic neurons reside both in the DRN and laterally within the adjacent periaqueductal gray (Belin et al., 1979; Allers and Sharp, 2003; Fu et al., 2010; Figure 1).

There are multiple cell types within the DRN, and these often have local axon collaterals. In addition to 5-HT cells and their intra-DRN axon projections (Bang et al., 2012) there are glutamate, GABA and dopamine neurons, and these neurotransmitters as well as 5-HT may be combined with different neuropeptides (Jolas and Aghajanian, 1997; Commons, 2009; Fu et al., 2010; Hioki et al., 2010). This, together with the multiple origins of glutamate and GABA inputs converging onto DRN neurons from extrinsic sources results in a highly complex DRN circuitry that remains still poorly understood. Only when we achieve a better understanding about how this rich set of afferents is organized within the DRN, could we determine what elements of the network are vulnerable to dysfunction in pathological states.

## High-resolution analysis of the DRN neuropil: glutamate excitatory drive

**Figure 2:**
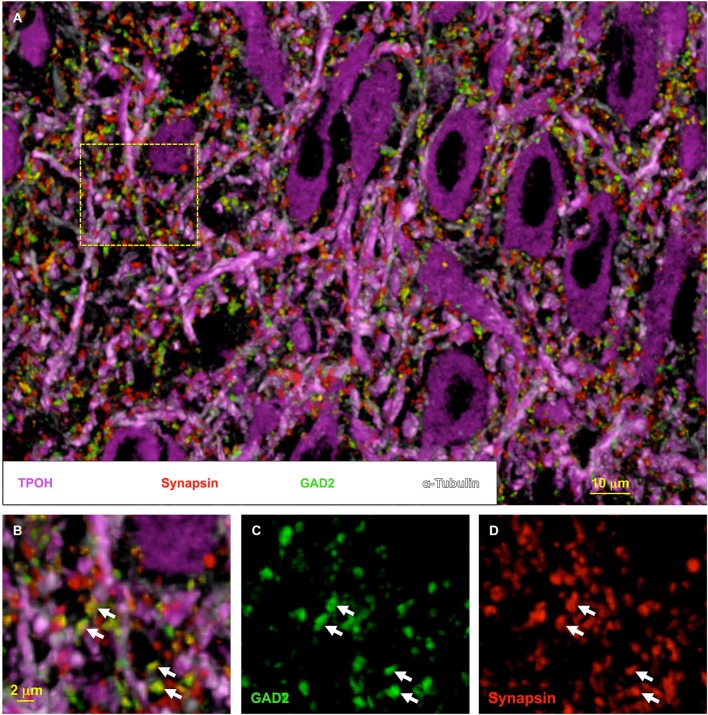
**High-resolution synaptic anatomy within the DRN using array tomography**. Volumetric image of the DRN rendered from multiple rounds of immunolabeling on 16 ultrathin (70 nm) serial-sections. In **(A)**, tryptophan hydroxylase (TPOH) labeling (magenta) reveals the high abundance of 5-HT cells in the nucleus. Additionally, the co-labeling with tubulin (light magenta) to identify the microtubule bundles indicates the compact distribution of their dendritic processes within the DRN neuropil. This organization is densely invaded by synaptic boutons identified by the presence of synapsin, a protein present at the synaptic vesicles (red). A proportion of these axon boutons are co-labeled for the GABA synthetic enzyme glutamate decarboxylase 65 (GAD2) (green) (indicated by the arrows in **(B–D)**). However, a large proportion of synaptic boutons do not contain the GABAergic marker, likely representing glutamate and other axonal populations within the DRN. Individually-resolved labeled puncta visualized with AT can be subjected to semi-automatic quantitative analysis using ImageJ software, giving a distribution of multiple populations of synaptic boutons within the same volume of tissue.

We recently applied a novel high-resolution immunofluorescence technique called Array Tomography (AT) to explore the organization of the DRN synaptic neuropil (Soiza-Reilly and Commons, 2011b; Soiza-Reilly et al., 2013). AT involves immunolabeling and imaging of ultrathin (70 nm) serial sections. Subsequently, the images can be reconstructed and rendered in 3D, and the relationships between the immunolabeled antigens can be visualized and quantitatively analyzed (Micheva and Smith, 2007; Micheva et al., 2010). One of the key features of AT is that it allows multiple rounds of immunolabeling/elution with a highly reliable preservation of antigens (Soiza-Reilly and Commons, 2011b). This results in high-resolution visualization of multiple antigens (typically 6–12) at the same time in 3D space (Figure 2). The physical sectioning of the tissue in AT (70 nm) provides a superb resolution especially in the *z*-axis, even beyond the theoretical limits dictated by Abbe’s law (Wang and Smith, 2012). Thin sectioning also eliminates issues related to antibody penetration, obtaining a more homogeneous distribution of the antibody on the section’s surface. Altogether these features allow mapping of multiple antigens in the same tissue volume, as well as the quantitative analysis of their spatial relationships to each other (Figure 2).

Using AT we examined the quantitative distribution of glutamate axons within the mouse DRN and determined the relative contribution of different glutamate axons arising from distinct populations of neurons to the excitatory drive of the DRN (Soiza-Reilly and Commons, 2011b). In our study, to avoid the potential inclusion of spurious immunolabeling in the quantitative analysis, we imposed a contingency such that the presence of a specific presynaptic marker for glutamate axons (e.g., VGLUT1-3) was analyzed with respect to a second and more general marker of glutamate synapses such as the postsynaptic protein PSD-95. Thus we found that synaptic boutons containing VGLUT2 provide the major glutamate input to the DRN when compared to those containing VGLUT1 or VGLUT3 (Soiza-Reilly and Commons, 2011b). Glutamate neurons in the prefrontal cortex predominantly express VGLUT1, while many subcortical regions sending axonal projections to the DRN preferentially express VGLUT2. These regions include many hypothalamic nuclei, the basal forebrain, as well as adjacent regions to the DRN such as the periaqueductal gray and parabrachial nucleus (Hisano et al., 2000; Fremeau et al., 2001; Herzog et al., 2001; Kaneko and Fujiyama, 2002; Kaneko et al., 2002; Varoqui et al., 2002). However, it still remains an open question how this balance between cortical vs. subcortical glutamate inputs influencing DRN neurons arises during development, to modulate the actions of 5-HT in the maturation of neural circuits (Gaspar et al., 2003), and whether faulty wiring of these circuits could contribute to the development of mental disorders that arise later in life such as depression or anxiety.

Additionally, in our study we did not find any preference in the cellular targets of these glutamatergic synaptic boutons and they were equally associated with both 5-HT as well as with non-5-HT neurons (Soiza-Reilly and Commons, 2011b). These findings are in agreement with previous ultrastructural studies using immunoelectron microscopy showing that glutamatergic synaptic innervations containing either VGLUT1 or VGLUT2 are not an exclusive feature of 5-HT neurons, and they are also associated with non-5-HT neurons (Commons et al., 2005). This is consistent at least with the dual influence of glutamate afferents arising from the prefrontal cortex upon 5-HT and GABA neurons within the DRN (Celada et al., 2001; Jankowski and Sesack, 2004).

However, previous studies suggested that 5-HT vs. non-5-HT neurons could be under the influence of different populations of glutamate axons or that these axons could have distinct control mechanisms. Specifically, a stress exposure appears to more selectively affect the activity of glutamatergic inputs to DRN 5-HT neurons than those associated with non-5-HT neurons (Kirby et al., 2007). Indeed, differential distribution of glutamate receptor subunits with respect to midline (5-HT-neuron rich) and lateral (GABA-neuron rich) locations in the DRN suggests different cell types may have different synaptic responses to glutamate (Soiza-Reilly and Commons, 2011a; Templin et al., 2012).

## Presynaptic interaction of glutamate and GABA transmission in the DRN

There was some functional evidence indicating a possible direct interaction between glutamate and GABA transmission in the DRN, influencing the activity of 5-HT cells (Kalén et al., 1989; Tao and Auerbach, 2003). However, the fine organization of glutamate and GABA synaptic innervations within the nucleus was poorly understood. Furthermore, the possible existence of synaptic arrangements that could underlie a direct interaction between glutamate and GABA transmission systems remained to be elucidated.

**Figure 3:**
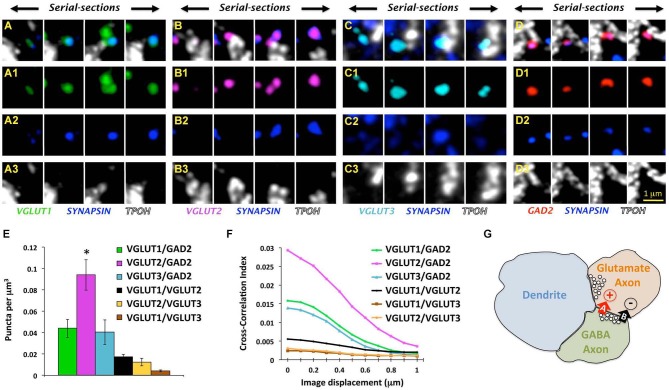
**Glutamate and GABA axon boutons in the DRN. (A–D)** Array tomographic visualization across four ultrathin (70 nm) serial-sections immunolabeled against VGLUT1-3 (green, magenta, and light blue, respectively) or GAD2 (red) to identify glutamate and GABAergic boutons, respectively. These immunodetected axon boutons are often co-labeled for synapsin (blue), and in some cases associated with TPOH-labeled dendrites (white). Adapted from Soiza-Reilly et al. (2013) reprinted with permission. **(E)** Total density of axo-axonic-like arrangements of four different types of axon boutons labeled either for VGLUT1-3 or GAD2 together with synapsin. The most abundant pair of axon boutons contained VGLUT2 and GAD2 ones (*p < 0.001 vs. either VGLUT1 or VGLUT3). Only a small proportion of axoaxonic-like arrangements were found between different glutamatergic boutons (e.g., VGLUT1/VGLUT2, VGLUT1/VGLUT3, and VGLUT2/VGLUT3). Bars represent SEM. Adapted from Soiza-Reilly et al. (2013) reprinted with permission. **(F)** Cross-correlation analysis of pixels between four different types of axon boutons using the ImageJ plugin JACoP (Bolte and Cordelières, 2006). This analysis indicated a higher degree of colocalization of VGLUT2 axon terminals with GAD2 boutons in comparison to other glutamate axons. Specifically, when imaging channels were aligned (*x* = at 0 μm of displacement), the pixels co-labeled for VGLUT1-3 and synapsin showed the highest value of correlation with pixels co-labeled for GAD2 and synapsin. When imaging channels were displaced with respect to each other all of the correlation values decreased indicating the spatial proximity of all three glutamate axon types with GABA boutons (Soiza-Reilly et al., 2013). Cross-correlations between different glutamate axon types were very low. **(G)** Schematic illustration of the GABAergic presynaptic modulation of glutamate release in the synaptic triads of the DRN. Adjacent glutamate and GABA axon terminals are closely associated with a postsynaptic dendrite that receives a typical excitatory asymmetric synapse from the glutamatergic bouton. GABA boutons would have the capacity of presynaptically influencing glutamate release from terminals through a dual mechanism involving a rapid/transient GABA-A receptor-mediated facilitation, and a more delayed and sustained GABA-B receptor inhibition.

Very recently, we tackled this problem using a combined approach involving AT, electron microscopy and electrophysiological recordings to examine the organization of GABA axons within the rat DRN as well as their possible association with glutamate axons (Soiza-Reilly et al., 2013). In that study, quantitative analysis of GABA axon boutons in the DRN as well as their spatial relationships to glutamate axons were performed primarily using AT. For that, we used immunolabeling against specific markers for glutamate (VGLUT1-3) and GABA (glutamate decarboxylase 65, GAD2) axons, together with a general marker for synaptic boutons such as the protein associated with synaptic vesicles synapsin 1. Thus, only axon boutons containing both a specific marker (e.g., VGLUT1-3 or GAD2) together with the general marker for synaptic boutons (i.e., synapsin) were included in the quantitative analysis (Figures 3A–D).

However, the absolute density of double immunolabeled objects could also include random instances depending on the abundance of antigens and specificity of labeling. We addressed this potential caveat by performing a cross-correlation analysis of pixels, providing insights about the specificity of spatial relationships between two populations of labeled objects (van Steensel et al., 1996; Micheva et al., 2010). Using this approach we validated the specificity of immunolabeled pairs VGLUT1-3 or GAD2 with synapsin, and we found that among them GABAergic and VGLUT2-containing glutamate axon boutons were about three times more prevalent than other axon types in the DRN. Additionally, we found their postsynaptic cellular targets included both 5-HT and non-5-HT neurons, with no apparent preference for one population over another (Soiza-Reilly et al., 2013).

Taking advantage of the capacity of AT for quantitative analysis we examined the possible presence of axo-axonic spatial arrangements involving glutamate and GABA axons in the DRN that could underlie a direct interaction between these neurotransmission systems. Using this approach we found that all three types of glutamate axon boutons (VGLUT1-3) were spatially related to GABA boutons, and among them relationships between GABA and VGLUT2-containing boutons were the most abundant (Figures 3E,F, Soiza-Reilly et al., 2013). This indicated that GABA axons in the DRN would interact with all the three types of glutamate axons according to their relative abundance in the nucleus, while associations between glutamate axons to each other were more rarely found (Figures 3E,F, Soiza-Reilly et al., 2013).

Complementary ultrastructural studies using immuno-electron microscopy for GAD2 in the DRN showed the presence of GABA axon boutons in close apposition to unlabeled axons with the morphology compatible with glutamatergic axon boutons (Soiza-Reilly et al., 2013). Further, these findings also indicated that they organize forming synaptic triads where both presynaptic GABA and glutamate axon boutons together associate with a single postsynaptic dendrite. Moreover, in these triads glutamate axon boutons were usually found establishing asymmetric-type synapses on the postsynaptic dendrite but not on GABA boutons (Soiza-Reilly et al., 2013). This raised the possibility that GABA could presynaptically modulate glutamate release in the DRN. We explored this hypothesis by studying the effects of GABA ligands on spontaneous miniature EPSCs in putative DRN 5-HT neurons using *in vitro* electrophysiological recordings. Indeed, we found opposite modulation of DRN glutamate synaptic transmission by GABA-A and GABA-B receptors. Specifically, GABA-A receptor activation facilitated glutamate release while GABA-B receptors on the contrary mediated an inhibitory effect (Soiza-Reilly et al., 2013). We hypothesize that this coincidental excitation and inhibition at the presynaptic glutamatergic terminal could operate similarly to that observed in the cerebellum (Pugh and Jahr, 2011), where a rapid transient GABA-A facilitation and a more delayed and sustained GABA-B inhibition have the capacity to temporally gate glutamate release in the DRN (Figure 3G).

Synaptic triads involving GABA and glutamate axons could at least partially explain previous pharmacological studies suggesting the interaction of these neurotransmitter systems to modulate 5-HT release (Tao and Auerbach, 2003). Moreover, the presynaptic modulation of glutamate transmission by GABA is also compatible with a possible role of GABA in state-dependent modulation of 5-HT system’s activity (Nitz and Siegel, 1997; Gervasoni et al., 2000). Additionally, since we found that all three types of glutamate axons can establish axo-axonic associations with GABA axons, this indicates that the presynaptic GABAergic modulation of glutamate transmission would represent a more general mechanism, influencing glutamate excitatory drives arising from multiple brain regions and converging to the DRN. Another important question that remains still open is whether glutamate and GABA synaptic inputs to the nucleus could be topographically organized to selectively modulate the activity of different subsets of DRN neurons.

From a GABA system perspective, only a proportion of GABA axon boutons were implicated in axo-axonic arrangements with glutamate axons. This raises the question of whether these GABA boutons arise from a particular subpopulation of GABAergic neurons located within or outside the DRN. Further studies combining the use of axonal tract-tracing with AT will give further insights into the selective role of GABAergic neurons in the presynaptic modulation of glutamate transmission in the DRN.

## Concluding remarks and future perspectives

The complete view of molecular and fine structural features of DRN synaptic circuits represents a stepping stone towards understanding of their role in normal and pathological neurotransmission, as well as if alterations in the structure of circuits could contribute to the pathophysiology of mental diseases. For this purpose, the use of recently developed high-throughput imaging techniques like AT has opened new avenues to characterize and better understand synaptic aspects of human neuropathology (Koffie et al., 2009; Hudry et al., 2013; Kay et al., 2013; Kopeikina et al., 2013). The unique ability of AT in providing high-throughput quantitative information about multiple synapse protein components co-existing in the same, unlimited in principle, volume of tissue, offers a wide spectrum of proteomic analyses of synapses. This makes possible not only to identify molecular signatures of synapses and classify them by studying the presence of neurotransmitter receptors and scaffold proteins (Micheva and Bruchez, 2012; O’Rourke et al., 2012), but also to determine the functional state of synapses by identifying channels, receptor active subunits as well as other regulatory proteins (Lacey et al., 2012). Additionally, the fact that AT allows the molecular characterization of synapses in their neuroanatomical context, it is possible to extract quantitative data about their involvement in synaptic motifs as well as their relationships to different cell types and subcellular compartments such as dendritic processes (Rah et al., 2013). Finally, AT is highly compatible with recently developed super-resolution microscopy techniques that have broken the diffraction limit (Micheva and Bruchez, 2012; Wang and Smith, 2012) such as the stimulated emission depletion microscopy (STED; Hell and Wichmann, 1994) or stochastic optical reconstruction microscopy (STORM; Rust et al., 2006). The combined use of these approaches allows the unique possibility of investigating with a superb resolution in all the optical planes (x-y-z axes), multiple molecular interactions within the same synapses in serially sectioned larger volumes of brain tissue. Moreover, the compatibility of AT with scanning electron microscopy allows to further locate synaptic features identified using fluorescence microscopy in their ultrastructural context (Micheva and Smith, 2007; Micheva et al., 2010). The use of these combined approaches will ultimately provide more comprehensive information about the molecular architecture of neural circuits particularly relevant in building more accurate wiring diagrams contributing to understanding of connectomes.

## Conflict of interest statement

The authors declare that the research was conducted in the absence of any commercial or financial relationships that could be construed as a potential conflict of interest.
